# In Vitro Cultures of *Schisandra chinensis* (Turcz.) Baill. (Chinese Magnolia Vine)—a Potential Biotechnological Rich Source of Therapeutically Important Phenolic Acids

**DOI:** 10.1007/s12010-012-9622-y

**Published:** 2012-03-08

**Authors:** Agnieszka Szopa, Halina Ekiert

**Affiliations:** Department of Pharmaceutical Botany, Jagiellonian University, Collegium Medicum, 9 Medyczna Str, 30-688 Kraków, Poland

**Keywords:** Schisandraceae, Shoot-differentiating callus culture, Undifferentiating callus culture, Chlorogenic acid, Protocatechuic acid, Salicylic acid

## Abstract

The contents of free phenolic acids and cinnamic acid were determined using an HPLC method in methanolic extracts from biomass of *Schisandra chinensis* (Turcz.) Baill. (Chinese magnolia vine) at different stages of organogenesis, cultured in vitro on a few variants of Murashige and Skoog (MS) medium, containing different concentrations of plant growth regulators 6-benzylaminopurine (BAP) and 1-naphthaleneacetic acid (NAA) (from 0.1 to 3.0 mg/l) and in extracts from overground parts of plants growing in vivo. Six of 12 analysed compounds were detected in all extracts: chlorogenic, *p*-coumaric, *p*-hydroxybenzoic, protocatechuic, salicylic and syringic acids. Total contents of the examined metabolites in biomass of shoot-differentiating callus culture cultivated on six MS medium variants were dependent on concentrations of growth regulators in the media and ranged from 14.90 to 60.05 mg/100 g d.w. Total contents of the compounds in biomass extracts from undifferentiating callus culture maintained only on two of six MS medium variants were higher and amounted to 74.54 and 78.24 mg/100 g d.w. Maximum total contents of phenolic acids in both types of in vitro cultures were greater than in fruits (55.73 mg/100 g d.w.) and leaves (4.55 mg/100 g d.w.) of plants gowning in vivo. Chlorogenic acid and salicylic acid were the main compounds identified in biomass extracts of shoot-differentiating callus cultures (max 22.60 and 21.17 mg/100 g d.w., respectively), while chlorogenic acid (max 38.43 mg/100 g d.w.) and protocatechuic acid (max 20.95 mg/100 g d.w.) prevailed in the extracts from undifferentiating callus cultures. Other compounds dominated in fruits, namely *p*-coumaric acid (23.36 mg/100 g d.w.) and syringic acid (14.96 mg/100 g d.w.). This is the first report on biochemical potential of cells from *S. chinensis* in vitro cultures to produce the biologically active phenolic acids. These are the first results on the analysis of this group of metabolites in overground parts of plants growing in vivo, too.

## Introduction

*Schisandra chinensis* (Turcz.) Baill (Chinese magnolia vine) is an East Asian species which currently is very popular in Europe as an adaptogenic plant [1]. We can find the monography of *Schisandrae chinensis fructus* in the newest edition of European Pharmacopoeia 7th and in the National Pharmacopoeias of European countries e.g. in Polish Pharmacopoeia 9th [2, 3]. This species has long been used in traditional Chinese medicine [4]. It was also much earlier known as a medicinal plant in North America than in Europe [5–7]. This plant species shows valuable biological activities. Apart from its adaptogenic properties, it is known as a hepatoprotectant, an antioxidant, and an anticancer drug. These properties have been attributed mostly to lignans, dibenzocyclooctadiene derivatives, which were identified in fruits, shoots, and leaves [5, 6, 8].

Other groups of active substances of the fruits were demonstrated to belong to organic acids (citric, malic, tartaric and fumaric acids), sugars, phytosterols (e.g. stigmasterol), tannins, volatile oil compounds (monoterpenes, sesquiterpenes), vitamins (e.g. C and E), and elements Cu, Mg, Ni and Zn [1, 5, 6]. There was not any information about the occurrence of phenolic acids in this plant species and other species of the *Schisandra* genus [8].

In vitro cultures of *S. chinensis* maintained in our laboratory have a potential to accumulate some lignans. Our studies analysing the contents of two main compounds of this group—schisandrol A and schisandrol B—revealed that biomass from shoot-differentiating callus culture could be a potential biotechnological source of these metabolites [9]. Maximum contents of both compounds obtained from in vitro cultures (about 70 and 86 mg/100 g d.w., respectively) were greater than in the studied leaves of the plants growing under natural conditions.

Lignans present in the plant species under study possess a unique structure, and their biogenesis has not been elucidated in detail, yet, though its initial steps are known to belong to the shikimic acid pathway, involved also in biogenesis of phenolic acids. These facts prompted us to undertake analysis of this group of compounds both in biomass cultured in vitro and in plants growing in vivo. The exceptional value of biological activity of this group of metabolites strengthened our decision of their investigation. Phenolic acids, either cinnamic or benzoic acid derivatives and depsides, like chlorogenic, and rosmarinic acids possess, e.g. anti-inflammatory, cholagogic, spasmolytic, hypolipemic, anti-aggregatory and immunostimulating actions [10–14]. Many new reports have also revealed anticancer, antiradical and antioxidant properties of some compounds belonging to this group, like protocatechuic acid and caffeic acid and depsides, e.g. chlorogenic acid [15–18]. Biotechnological studies of *S. chinensis* carried out in other scientific centres focused on possibility to use enzymatic potential of the cells cultured in vitro for biotransformation of hydroquinone into phenolic glycosides. Few papers dealt also with micropropagation of this plant species, mainly by somatic embryogenesis. One report addressed the problem of validation of lignans assays in material from in vitro cultures [19–23].

The present study aimed to investigate the accumulation of free phenolic acids and cinnamic acid in biomass from in vitro cultures at different stages of differentiation cultivated on several variants of Murashige and Skoog (MS) medium [24] containing varying amounts of plant growth regulators: 6-benzylaminopurine (BAP; cytokinin) and 1-naphthaleneacetic acid (NAA; auxin) ranging from 0.1 to 3.0 mg/l of the medium and to compare biosynthetic capacities of the cells from in vitro cultures with cells of plants growing in vivo. Moreover, the studies aimed to demonstrate the utility of in vitro cultures of *S. chinensis* as a potential biotechnological source of the biologically active phenolic acids.

Twelve compounds were determined by an HPLC method in methanolic extracts from biomass cultured in vitro and in the extracts from overground parts of plants growing under natural conditions (fruits, leaves). In vitro cultures of *S. chinensis* have not been studied so far in terms of the biochemical potential for production of free phenolic acids. Until now the native plant has not been studied for the contents of these metabolites, too. We found only interesting information, about the high amounts of cinnamic acid—precursor of one group of phenolic acids, in leaves of these plant species [25].

## Materials and Methods

### Establishment of In Vitro Cultures

The in vitro cultures were established from leaf buds of *S. chinensis* (Turcz.) Baill. (Schisandraceae) from the Rogów Arboretum—Warsaw University of Life Sciences, Forest Experimental Station in Rogów (Poland); for details, see Szopa and Ekiert [9]. After 4 weeks we obtained two different cultures: shoot-differentiating callus culture and undifferentiating callus culture.

### Experimental In Vitro Culture

The shoot-differentiating callus culture was maintained on six solid MS [24] medium variants differing in concentrations of plant growth regulators, BAP and NAA (in milligrams per litre): 0.1 and 2.0, 0.5 and 2.0, 2.0 and 0.5, 2.0 and 1.0, 2.0 and 2.0, and 3.0 and 1.0. The callus culture was cultivated on two solid MS medium variants, containing BAP and NAA (in milligrams per litre): 2.0 and 2.0, and 3.0 and 1.0. Both types of cultures were grown under constant artificial light (4 W/m^2^, LF-40W lamp, daylight, Piła) at 25 ± 2 °C for 4 weeks (three series).

### Plant Material

Plant material harvested in Poland in 2010, analysed for comparison, comprised fruits and leaves of the plants growing under natural conditions (Rogów Arboretum—Warsaw University of Life Sciences, Forest Experimental Station in Rogów, Poland).

### Extraction and HPLC Analysis

The dried biomass from both types of in vitro cultures collected after 4-week growth cycles (three series) and plant material—fruits and leaves (from 0.2 to 0.5 g)—were extracted twice with boiling methanol for 3 h. In the methanolic extracts, chromatographic quantification of 11 phenolic acids and cinnamic acid was performed with the HPLC method according to Tian et al. [26]. Separation was performed using an analytical column Kinetex™ 18C (150 × 4.6 mm, 2.6 μm) at 25 °C. The mobile phase consisted of 0.1 % trifluoroacetic acid (A) and acetonitrile (B); a flow rate was set on 1.0 ml/min (gradient programme), injection volume 5 μl. Detection wavelength was set at 254 nm. Quantification was made by comparison with standards: caffeic, chlorogenic, cinnamic, protocatechuic, rosmarinic, salicylic, sinapic and syringic acids (Sigma), and *p*-coumaric, ferulic, *p*-hydroxybenzoic, and vanillic acids (Fluka).

## Results

### Shoot-Differentiating Callus Culture

Dry biomass of shoot-differentiating callus culture increased 2.1–6.1-fold during 4-week growth cycles on the tested six variants of MS medium. High over fivefold biomass increments were observed on the medium variants containing cytokinin (BAP) and auxin (NAA) at concentrations of 2 and 2, 2 and 1, and 3 and 1 mg/l of the medium.

**Fig. 1:**
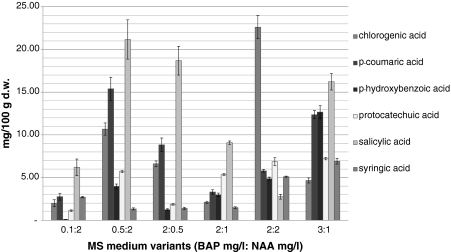
Contents (in milligrams/100 g d.w.) of phenolic acids in biomass extracts from *S. chinensis* shoot-differentiating callus culture. The values are the means of three experiments ± SE

Among 12 compounds under study, six phenolic acids were identified in the extracts: chlorogenic, *p*-coumaric, *p*-hydroxybenzoic, protocatechuic, salicylic and syringic acids. None of the extracts under analysis contained caffeic, cinnamic, ferulic, rosmarinic, sinapic and vanillic acids (Fig. 1).

Total content of phenolic acids was dependent on MS medium variant and ranged from 14.90 to 60.05 mg/100 g d.w. Three medium variants containing 3 mg/l BAP and 1 mg/l NAA, 0.5 mg/l BAP and 2 mg/l NAA, and 2 mg/l BAP and 2 mg/l NAA were demonstrated to be beneficial for accumulation of the studied metabolites (total contents reached 60.05, 58.25 and 47.98 mg/100 g d.w., respectively; Table 1).

**Table 1:** Total contents (in milligrams/100 g d.w.) of free phenolic acids in biomass extracts from two types of *S. chinensis* in vitro cultures cultured on variants of MS medium, containing different concentrations of plant growth regulators

Type of culture	MS medium variants [BAP (mg/l):NAA (mg/l)]					
	0.1:2	0.5:2	2:0.5	2:1	2:2	3:1
Shoot-differentiating callus culture	14.90	58.25	38.65	24.34	47.98	60.05
Undifferentiating callus culture	–	–	–	–	78.24	74.54

Undifferentiating callus culture was maintained only on two variants of MS medium (see Materials and methods)

The contents of individual metabolites were very diverse and ranged from 0.1 to 22.60 mg/100 g d.w. Their concentrations changed from 4.03- to 126.30-fold depending on MS medium variant (Fig. 1).

Maximum contents of protocatechuic acid and syringic acid amounted to about 7.00 mg/100 g d.w. Maximum amounts of *p*-hydroxybenzoic acid were almost two times higher reaching about 13.00 mg/100 g d.w. The accumulation of these three metabolites was enhanced on the MS medium variant containing 3 mg/l BAP and 1 mg/l NAA. Two compounds, chlorogenic acid and salicylic acid, were accumulated at higher amounts (max about 22.00 mg/100 g d.w.; Table 2). The greatest content of chlorogenic acid was observed in biomass cultured on the MS medium variant supplemented with 2 mg/l BAP and 2 mg/l NAA. It is worth emphasizing that high amounts of salicylic acid (over 16.00 mg/100 g d.w.) were accumulated on three MS medium variants (max content on the medium containing 0.5 mg/l BAP and 2 mg/l NAA).

**Table 2:** Contents (in milligrams/100 g d.w.) of free phenolic acids in fruits and leaves of *Schisandra chinensis* growing in vivo and their maximal contents in biomass from in vitro cultures

Phenolic acids	Fruits	Leaves	Shoot-differentiating callus culture	Undifferentiating callus culture
Chlorogenic acid	1.98	1.37	22.60	38.43
*p*-Coumaric acid	23.36	1.27	15.39	7.28
*p*-Hydroxybenzoic acid	2.60	0.20	12.66	4.43
Protocatechuic acid	5.81	0.33	7.22	20.95
Salicylic acid	7.01	0.92	21.17	0.67
Syringic acid	14.96	0.46	6.92	9.51
Total content	55.73	4.55	60.05	78.24

### Undifferentiating Callus Culture

Undifferentiating callus culture was maintained on two variants of MS medium containing 2 mg/l BAP and 2 mg/l NAA, and 3 mg/l BAP and 1 mg/l NAA. These media were chosen because the highest biomass increments in shoot-differentiating callus cultures of *S. chinensis* were observed at these concentrations of plant growth regulators.

**Fig. 2:**
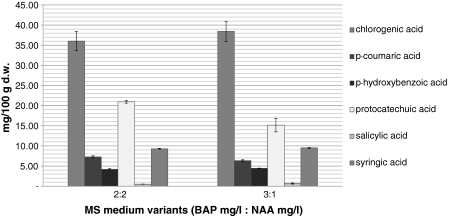
Contents (in milligrams/100 g d.w.) of phenolic acids in biomass extracts from *S. chinensis* undifferentiating callus culture. The values are the means of three experiments ± SE

Biomass increments in undifferentiating callus cultures maintained on the above-mentioned two variants of MS medium were of the same order of magnitude and amounted to 7.4- and 7.6-fold, respectively. The extracts under analysis contained also six metabolites of 12 compounds determined chlorogenic, *p*-coumaric, *p*-hydroxybenzoic, protocatechuic, salicylic and syringic acids (Fig. 2). These phenolic acids were the same as those detected in biomass extracts of shoot-differentiating callus cultures. Maximum total contents of the mentioned six compounds were 78.24 and 74.54 mg/100 g d.w., respectively, and were 1.24- and 1.63-fold higher in comparison with the respective values assayed in the extracts from biomass of shoot-differentiating callus cultures (Table 1).

Chlorogenic acid and protocatechuic acid were the main metabolites (max contents 38.43 and 20.95 mg/100 g d.w., respectively; Fig. 2). The amounts of syringic acid (about 10.00 mg/100 g d.w.), *p*-coumaric acid (about 7.00 mg/100 g d.w.) and *p*-hydroxybenzoic acid (about 4–4.5 mg/100 g d.w.) were smaller. Salicylic acid was accumulated at the lowest amounts (below 0.7 mg/100 g d.w.; Table 2). The contents of individual metabolites accumulated on two MS medium variants under study were generally comparable. Only in the case of protocatechuic acid, the medium containing 2 mg/l BAP and 2 mg/l NAA was more conducive to accumulation of this compound.

## Discussion

Concentrations of growth regulators in the MS [24] medium variants had a significant effect on the accumulation of free phenolic acids in shoot-differentiating callus cultures of *S. chinensis*. Depending on the MS medium variant, total contents of the compounds under study ranged from 14.90 to 60.05 mg/100 g d.w. whereas contents of individual metabolites changed from 4.03- to 126.30-fold.

The effect of composition and contents of growth regulators in culture media on the accumulation of secondary metabolites in in vitro cultures is well known [27]. Our earlier studies demonstrated their influence on the accumulation of different groups of metabolites: linear furanocoumarins in callus cultures of two Apiaceae species—*Ammi majus* and *Pastinaca sativa* [28, 29], free phenolic acids in shoot cultures of *Ruta graveolens* [30] and recently lignans in shoot-differentiating callus cultures of *S. chinensis* [9].

In the present studies, maximum total contents of the compounds in shoot-differentiating callus culture were obtained on MS media containing 3 mg/l BAP and 1 mg/l NAA, 0.5 mg/l BAP and 2 mg/l NAA, and 2 mg/l BAP and 2 mg/l NAA. The first of the above-mentioned medium was also the best medium for lignans accumulation in shoot-differentiating callus culture of *S. chinensis* [9].

Two media proposed now as beneficial for the production of phenolic acids in *S. chinensis* in vitro cultures (3 mg/l BAP and 1 mg/l NAA, and 2 mg/l BAP and 2 mg/l NAA) were also favourable for biomass growth (five- to sixfold increase in dry biomass). These variants of MS medium can be proposed as universal, both “productive” and “growth-promoting” media. Undifferentiating callus cultures were tested only on two MS medium variants containing 3 mg/l BAP and 1 mg/l NAA, and 2 mg/l BAP and 2 mg/l NAA. Total contents of phenolic acids obtained on these media were 1.24- and 1.63-fold greater, respectively, than in the extracts from shoot-differentiating callus cultures (Table 1). This result is surprising because usually a higher degree of differentiation and organogenesis promotes the accumulation of secondary metabolites [31]. Our earlier experiments on lignans accumulation in *S. chinensis* in vitro cultures demonstrated many times higher contents of the lignans schisandrol A and B in shoot-differentiating callus culture (about 70 and 86 mg/100 g d.w.) than in undifferentiating callus culture (below 3 mg/100 g d.w.) [9]. As indicated by our studies, the accumulation of phenolic acids was not linked with the high degree of differentiation of *S. chinensis* biomass cultured in vitro [32].

Chlorogenic acid was accumulated in shoot-differentiating callus culture at marked amounts, and in undifferentiating callus culture, its contents were maximal (Table 2). This compound is a product of initial steps of the shikimic acid pathway and is accumulated in many plant species [32] and in in vitro cultures of some plant species (e.g. *Arnica montana*, *Eleutherococcus senticosus*) [12]. Therefore, it is not surprising that this metabolite was accumulated at high amounts in the in vitro cultures under study. Maximum contents of chlorogenic acid obtained from the biomass cultured in vitro (reaching almost 20 and 40 mg/100 g d.w. in biomass at different stages of organogenesis) are interesting from practical point of view. These results are very promising especially considering exceptional biological activity of chlorogenic acid (e.g. its anticancer, antiradical and immunostimulating properties) [15–18].

Extracts from overground parts of plants growing in vivo contained the same compounds under study as biomass from in vitro cultures (Table 2). Extracts of fruits, which are a pharmaceutical raw material, contained *p*-coumaric acid (23.36 mg/100 g d.w.) and syringic acid (14.96 mg/100 g d.w.) as dominating metabolites. Chlorogenic acid content was very low (below 2 mg/100 g d.w.). Leaves turned out to be a very poor source of phenolic acids (total contents approximated 4.5 mg/100 g d.w.; Table 2). In leaves extracts, chlorogenic, *p*-coumaric and salicylic acids were the main metabolites (1–1.4 mg/100 g d.w.). Two of these compounds dominated in biomass extracts from in vitro cultures, too. Maximum total contents of phenolic acids in biomass from in vitro cultures were 1.4 and 17.2 times higher than in overground parts of plants—fruits and leaves, respectively (Table 2). Already at the present stage of research, both types of *S. chinensis* in vitro cultures can be proposed as a rich potential biotechnological source of biologically active phenolic acids and a good model for further studies on optimization of production of these metabolites.

The results presented for the first time the biochemical potential of cells from *S. chinensis* in vitro cultures to produce the phenolic acids. These are the first results on the analysis of this group of metabolites in overground parts of plant growing in vivo, too.
